# Engagement in Human-Agent Interaction: An Overview

**DOI:** 10.3389/frobt.2020.00092

**Published:** 2020-08-04

**Authors:** Catharine Oertel, Ginevra Castellano, Mohamed Chetouani, Jauwairia Nasir, Mohammad Obaid, Catherine Pelachaud, Christopher Peters

**Affiliations:** ^1^Interactive Intelligence, Intelligent Systems, EWI, Delft University of Technology, Delft, Netherlands; ^2^Uppsala Social Robotics Lab, Department of Information Technology, Uppsala University, Uppsala, Sweden; ^3^Institute for Intelligent Systems and Robotics, CNRS UMR 7222, Sorbonne University, Paris, France; ^4^Computer-Human Interaction in Learning and Instruction Lab, École Polytechnique Fédérale de Lausanne, Lausanne, Switzerland; ^5^Interaction Design Division, Department of Computer Science and Engineering, Chalmers University of Technology, Gothenburg, Sweden; ^6^CNRS, Institute for Intelligent Systems and Robotics, Sorbonne University, Paris, France; ^7^Embodied Social Agents Lab (ESAL), School of Electrical Engineering and Computer Science, KTH Royal Institute of Technology, Stockholm, Sweden

**Keywords:** engagement, human-robot interaction (HRI), human-agent interaction (HAI), engagement perception, engagement generation

## Abstract

Engagement is a concept of the utmost importance in human-computer interaction, not only for informing the design and implementation of interfaces, but also for enabling more sophisticated interfaces capable of adapting to users. While the notion of engagement is actively being studied in a diverse set of domains, the term has been used to refer to a number of related, but different concepts. In fact it has been referred to across different disciplines under different names and with different connotations in mind. Therefore, it can be quite difficult to understand what the meaning of engagement is and how one study relates to another one accordingly. Engagement has been studied not only in human-human, but also in human-agent interactions i.e., interactions with physical robots and embodied virtual agents. In this overview article we focus on different factors involved in engagement studies, distinguishing especially between those studies that address task and social engagement, involve children and adults, are conducted in a lab or aimed for long term interaction. We also present models for detecting engagement and for generating multimodal behaviors to show engagement.

## 1. Introduction

**Figure 1:**
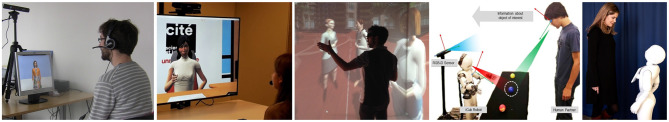
Examples of virtual and physical agents in typical engagement scenarios with humans.

**Figure 2:**
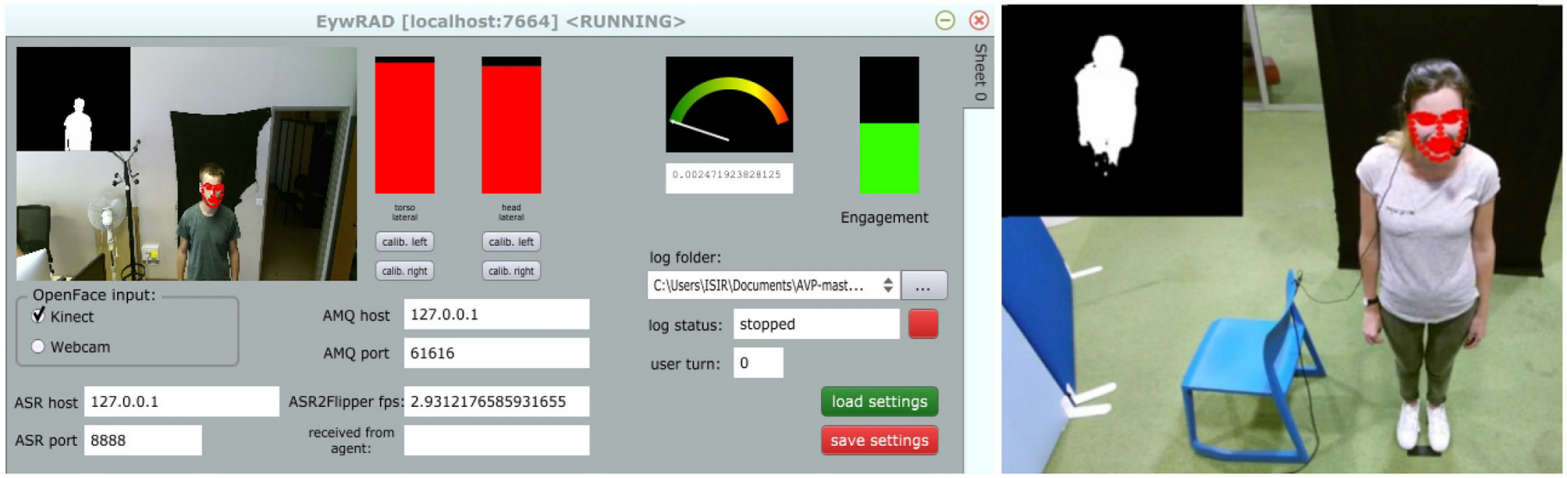
Examples of engagement detection systems.

Engagement is a concept of the utmost importance in human-machine interaction, not only for informing the design and implementation of interfaces, but also for enabling more sophisticated interfaces capable of adapting to users. This is particularly true when the interface is an agent (see Figure 1), be it virtual or robotic, that converses with human users. In the former case, the agents detect users' engagement (see Figure 2) while in the latter case the agents adapt to the detected engagement. These agents all have a common goal, namely to have users continue interacting with them and thus manage users' engagement in the interaction. Thus, for human-agent interaction engagement, both perception and generation are important issues. Perceiving how engaged users are can be beneficial information for adapting agent behavior. It can also be a sign of the quality of the interaction and user's experience with the system. Similarly generating engaged behaviors in an agent can be beneficial for human-perception in terms of social awareness.

The term engagement is being used across a number of diverse research domains, both scientific and commercial. Its definition and use varies considerably and can be confusing, especially for researchers approaching the topic for the first time. In fact engagement recently gained increasing popularity, in particular with the development of interaction paradigms between humans and embodied agents, such as virtual characters and robots (see for example Leite et al., 2016). Yet, there remains great variability, overlap and often vagueness with respect to the definition of engagement. It is often used synonymously to refer to a number of related concepts, such as interest, sustained attention, immersion and involvement. Recently several papers attempted to provide definition(s) of engagement. Researchers have also proposed computational models to compute engagement, both to analyse a human's level of engagement and to drive agent's behavior to show its engagement. See Sanghvi et al. (2011) and Sidner et al. (2003) for respective examples. The models vary in terms of definition of engagement (which phenomenon is modeled) and of expressive manifestation (which multimodal behaviors are involved). The fact that more and more papers are trying to provide an overview of engagement is warranted by the great diversity of definitions of engagement across different papers. *The purpose of this article is to provide insights into the use of engagement, particularly as it pertains to human-agent interaction, with a focus on embodied agents such as embodied conversational agents and social robots*.

In this article, we review the literature with the aim of answering the following questions:

1. Specifically for human-agent interaction, what are the engagement definitions most commonly used and how do they differ from one another?
2. How does the definition of engagement and its implementation differ along several factors such as interaction settings (real world or laboratory), interaction types (short or long interaction), interaction goals (social interaction or task performance), and user types (adults or children)?
3. How are engagement annotations being conducted? Which methods and features are being used to detect engagement and which expressions are being used to generate engagement behaviors?
4. What are the functions of engagement and which adaptation strategies are applied by the agent in order to maintain it, increase it or show disengagement?

The structure of the paper is as follows. First we present our method to gather articles in the literature. We present the annotation schema we follow to analyse and cluster these papers. In section 3, we list the various definitions of engagement found in the selected papers. From the next section onward, each section focuses on specific aspects of engagement. In section 4, we review how engagement is defined through the different scenarios that are commonly used in human-agent interaction. Section 5 focuses on computational models to detect engagement while section 6 describes the models to drive agent's behaviors to display engagement. Section 7 considers the additional issues and requirements when humans are to engage in sustained interaction over long periods of time with artificial systems. Since many of the studies considered here have adult participant groups, in section 8 we specifically report those studies that involve children. Finally, we conclude by highlighting gaps in the literature and point to possible future research.

## 2. Method

We started curating a list of relevant papers, in November 2017, by doing a search query using the terms “engagement+human-robot interaction” in Scopus and Google Scholar, two popular citation databases. Thereafter, to consolidate a full list of any newly published articles, the search was repeated every 6 months up until December 2019. The curated list of papers went through the following inclusion criteria:

- Is the paper covering the topic of engagement in human-agent interaction, including detecting it and generating behavior to manifest engagement? This criterion entails that papers can include robotic agents or virtual agents.
- Is the paper over four pages in length? With this criterion, the inclusion of abstracts or poster publications is eliminated.
- Is the content of the paper not overlapping significantly with another paper from the same author(s)? If the paper does overlap, then the most elaborated paper is selected for inclusion.

The initial curated list resulted in 189 papers in total and, based on the inclusion criteria above, 20 were excluded resulting in a final set of 169 papers. Based on a preliminary review of the 169 papers and the questions enumerated in the Introduction section, we developed an annotation schema (see Table 2) to allow us investigate and answer each of the questions.

Using the selected 169 relevant articles, we conducted a full review on each paper to extract the details of the annotation schema categories presented in Table 2. The procedure commenced with an assignment of annotation task to authors of this paper. During the process, the annotators discussed and resolved any ambiguous statements in papers that relate to any of the annotation categories. In order to facilitate the reviewing process and to guarantee reproducibility, a shared spread-sheet was created containing all annotated data. The schema categories represent factual data extracted from each paper. While all 169 papers contribute to the general overviews, discussions and statistics in this survey, due to space constraints, it is not possible to report in detail on all of them. Therefore, all papers are included in the references section, but only a subset of those papers are cited when they have been discussed in more detail.

### 2.1. Statistics

**Figure 3:**
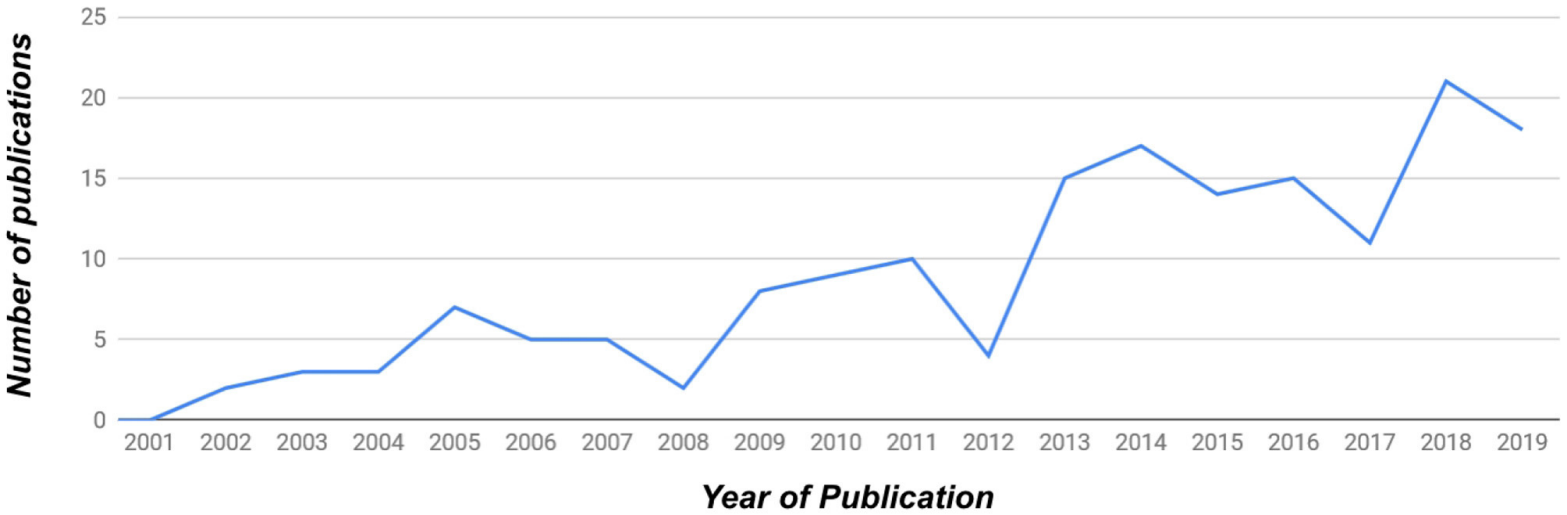
The distribution of publications per year covered in this survey. A total of 169 publications were considered in total between the years 2001 and 2019.

See Figure 3 for a graph of the number of publications covered in this survey according to year. Overall, a total of 169 publications were considered between the years 2001 and 2019. Of the 169 papers, 139 concerned a physical robot (88 papers, i.e., 52% of overall papers) and/or virtual agent (51, 30%) embodiment. 39 (23%) papers involved studies that included or focused on children as participants.

**Figure 4:**
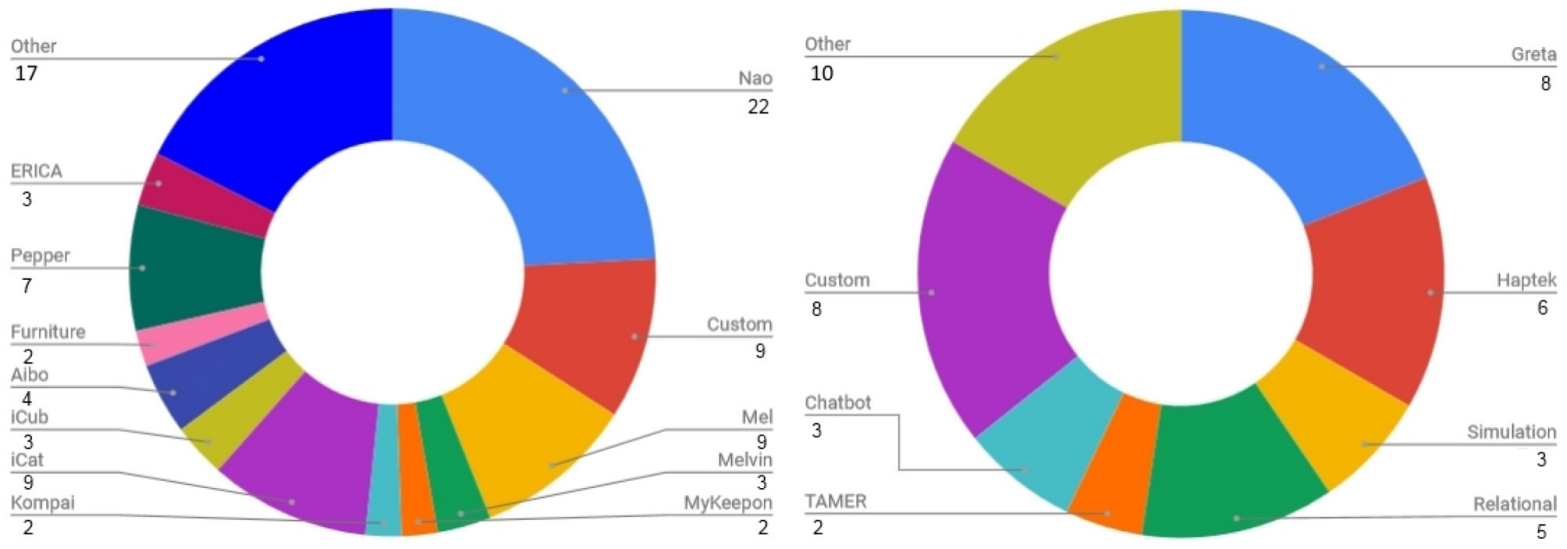
Overview of number of mentions of specific **(Left)** robot types and **(Right)** virtual agent types used in studies. Note that some studies involved the use of multiple robot/agent types, while others did not use any robot or virtual agent, or did not specify the type involved.

**Figure 5:**
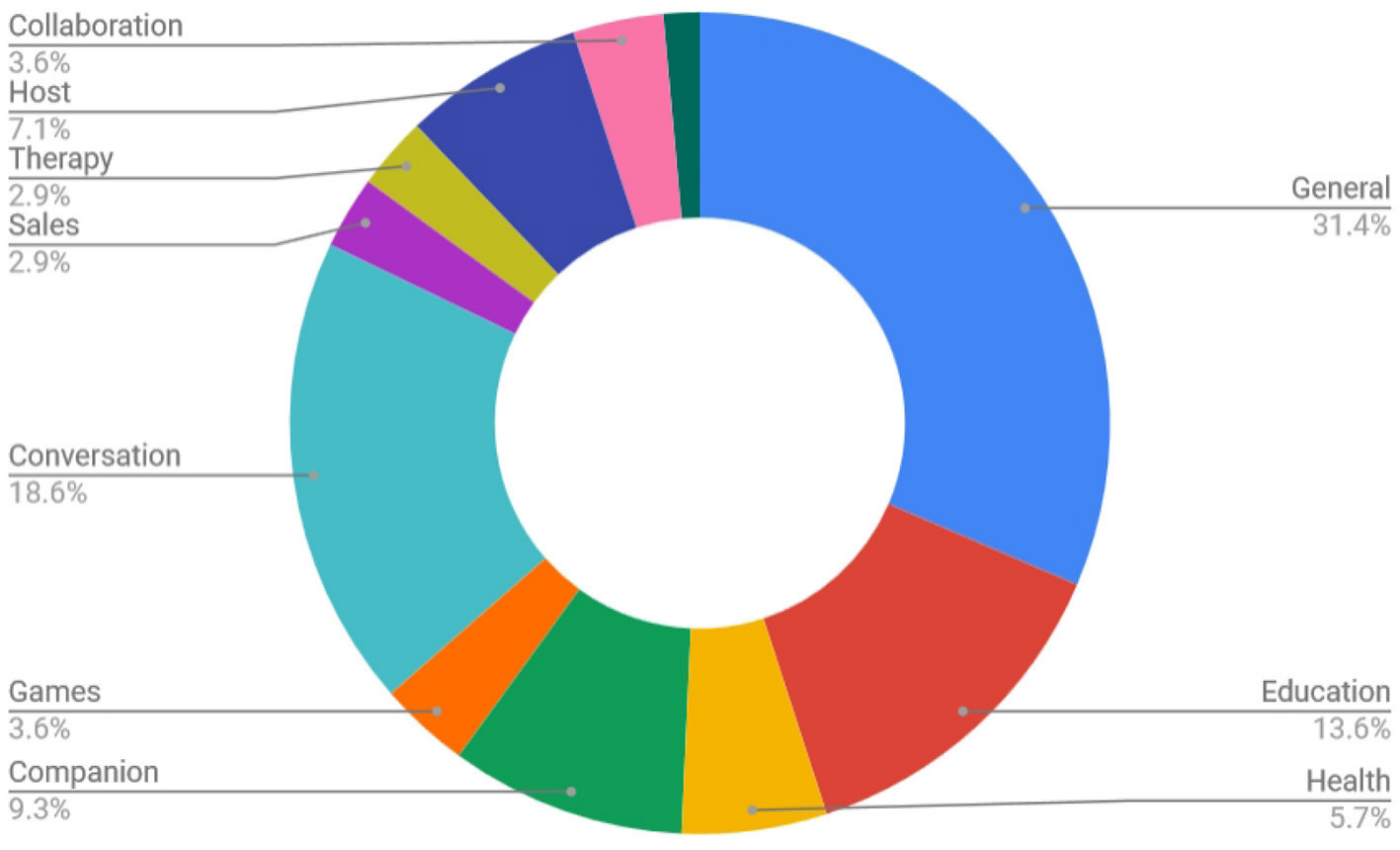
Overview of all publications in this survey according to application type when it was specified (140 in total specified, 29 unspecified, or could not be identified).

**Figure 6:**
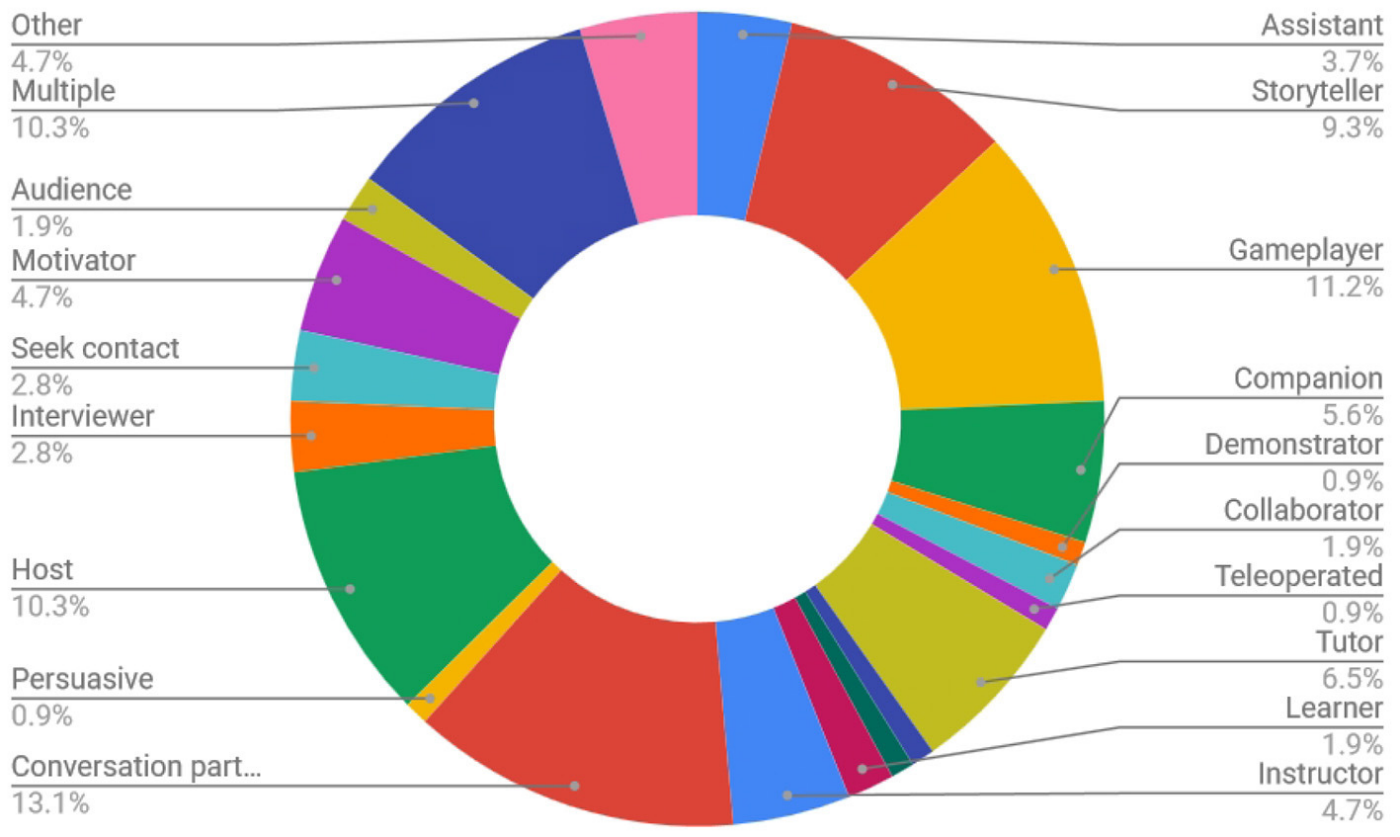
Overview of all publications in this survey according to the role of the robot or virtual agent when it was specified (107 in total specified, 62 unspecified, or could not be identified).

Papers were categorized according to the application area of the paper, the role of the agent/robot, type of robot, the number of participants in the study and the age of participants (adults or children). For each specific category, labels were enumerated by surveying all included papers and then maintaining those labels that appeared in more than one paper. See Figure 4 for a graph of the different robot embodiments used in studies covered in this survey. The labels consisted of a type or name of robot or virtual agent if more than one study took place with it. Otherwise the label *other* was used. *Greta, Relational*^1^, *Haptek* and *TAMER* refer to specific virtual agent solutions or frameworks, while *Chatbot, Simulation, Custom*, and *Other* are categories. A graph of the applications related to engagement covered in the papers in this survey is presented in Figure 5. They cover the categories of *education, health, games, companion, conversation, sales, therapy, host*, and *collaboration*. See Figure 6 for a graph of the role of the agent. The role category included the labels *assistant, storyteller, game opponent, demonstrator, collaborator, teleoperated, tutor, therapist, elicitor, entertainer, learner, instructor, conversation partner, persuader, host, interviewer, contact seeker, motivator, audience, multiple* (for multiple categories) and *other*. A few of the less well-known roles can be better explained by examples; for instance, in Anzalone et al. (2015), the robot is used as an *elicitor*, i.e., it elicits certain behaviors in humans in various face-to-face interaction scenarios. Furthermore, in Rani and Sarkar (2005), a *teleoperated* robot detects the engagement levels of its operator through physiological sensors and adapts its behavior accordingly. Lastly, in Baek et al. (2014), where the robot acts as a *contact seeker*, a study is conducted to explore how a communicator type (human, robot, product) impacts social presence and shyness of participants when they come in physical contact with each of them.

### 2.2. Evaluation Methodology

**Figure 7:**
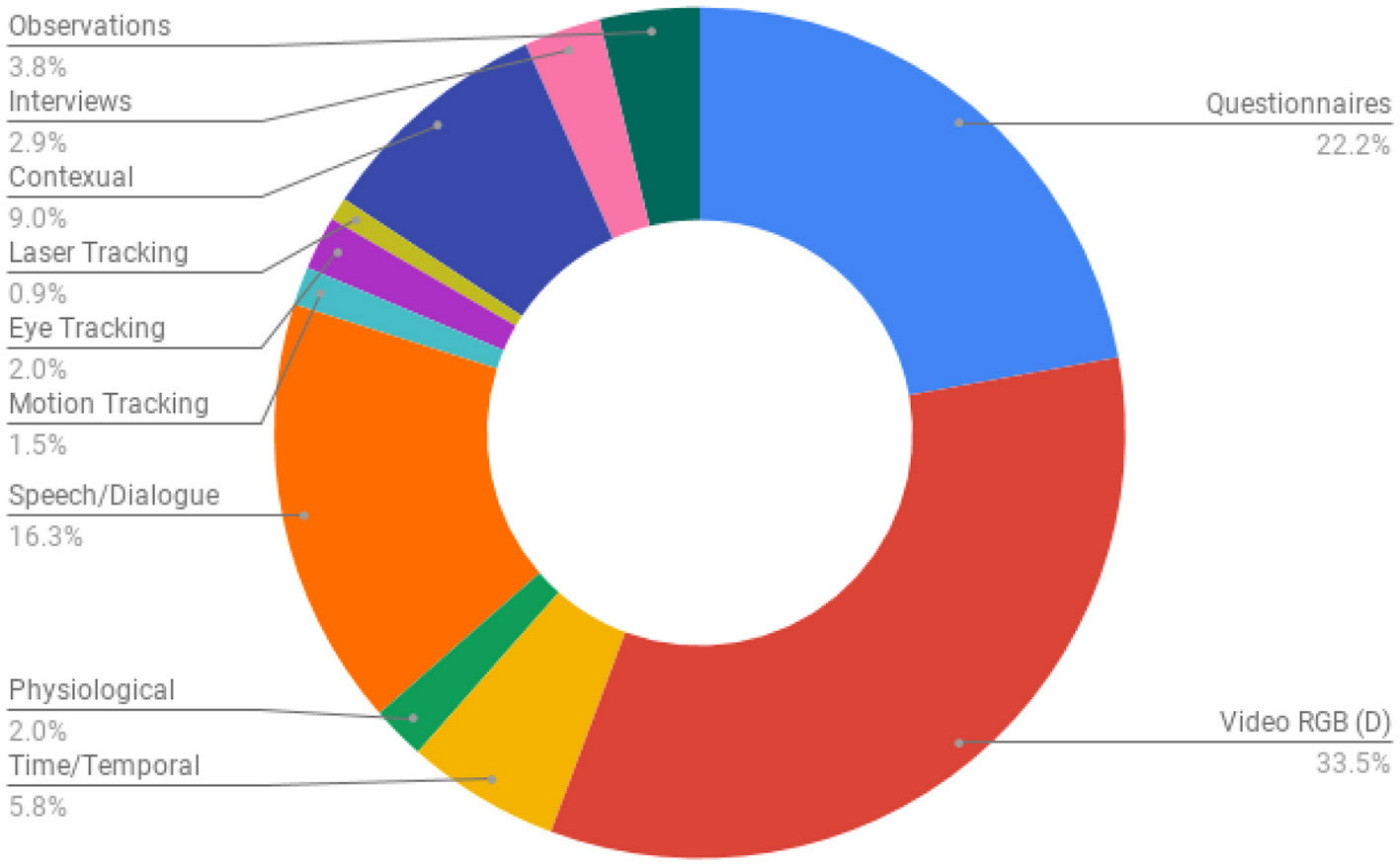
The type of data collected in the evaluation studies conducted in the curated list of papers. Note that many studies collected multiple data types while some did not collect any.

To understand how evaluations in the curated list of papers investigated the users' perception in their work, we extracted the type of evaluation data collected in each of the papers. After an initial review of all the papers, it was apparent that 23 papers did not include an evaluation study and were found to be focused on technical, modeling or conceptual contributions. For the remaining papers, we found thirteen types of evaluation tools, both objective and subjective, to measure the users' perception in the presented works. The data types reported include questionnaires, RGB video recordings, depth camera recordings, time/temporal performances, post study interviews, observations, physiological sensor data, tracking sensor technologies (motion, eye, and laser tracking), speech and dialogue recording, and contextual and application records (such as game scores, number of moves, implicit touch gestures, logs etc). Figure 7 represents the percentage of each type used in the curated list of papers.

It is apparent from Figure 7 that authors approached measuring the users' perception in HRI engagement research mainly through video analysis (33.5%), speech/dialogue analysis (16.3%), contextual and application performance data (9.0%), and questionnaires (22.2%).

## 3. Definition of Engagement

As previously mentioned, there is great variation concerning definitions of engagement. Papers can be divided into those that consider engagement as a process and those that treat engagement as a state. The state point of view assumes that one is either engaged or not engaged (e.g., Inoue et al., 2018), while the process point of view assumes that there are different processes that unfold during an interaction. Here the action of getting engaged is part of the construct of engagement itself. The most commonly used example of a process definition is in Sidner et al. (2003), which defines engagement as “the process by which interactors start, maintain, and end their perceived connections to each other during an interaction.” Examples of studies which are using this definition are Holroyd et al. (2011), Bohus and Horvitz (2009b), Alami et al. (2005), Sidner et al. (2006), Nakano and Nishida (2005), and Anzalone et al. (2015). There are also those who slightly adapt or alter the definition. One example is Bohus and Horvitz (2009a). Their definition of engagement is “the process subsuming the joint, coordinated activities by which participants initiate, maintain, join, abandon, suspend, resume, or terminate an interaction.” It also includes the concepts of abandon, suspend, and resume.

A second distinction can be made depending on who or what is the receiver of user engagement. For example, in human-agent interaction, the human user can be engaged with the agent (and vice-versa), the task that user and agent are be involved in, or the whole system (i.e., agent and task). The former case is often called *social engagement* and the next one *task engagement*.

Regarding the definition of engagement in a conversational setting, Coker and Burgoon (1987) were the first, to our knowledge, who attempted a definition. They are referring to a concept called *conversational involvement* which, for all intents and purposes of this paper, refers to the same concept as engagement. They defined four distinct variables: “the degree of animation and dynamism,” “the tendency to be interested in, attentive to, and adaptive to the other in a conversation,” the “immediacy” in the behavior of the interlocutors, and their degree of “social anxiety.”

Engagement with an agent is typically referred to as *social engagement*. There is variation in the definition of social engagement. It can be defined as any interaction a human has with either another human being or a robot (Sidner et al., 2003; Poggi, 2007). Another definition of “social engagement” is provided by Moshkina et al. (2014) as “a core social activity that refers to an individual's behavior within a social group.” The commonality which can be highlighted between both of these two definitions is that social engagement happens in interaction with one another. These definitions remain relatively vague and leave space to encompass a great variety of activities and experimental set-ups with different degrees of socialness. Within the studies reviewed in this article, the activities that could be classified as being more social include storytelling (see for example Szafir and Mutlu, 2012), followed by unstructured conversations and games. While unstructured conversations can encompass many aspects of conversation, generally they are not task-driven.

Social engagement very often also includes an affective component (see for example Corrigan et al., 2016; Biancardi et al., 2019a; Sohail et al., 2019; Youssef et al., 2019). The definition of the affective component of engagement often remains vague. It is sometimes related to fun, as is the case in Rehm and Jensen (2015), entertainment, as exemplified in Vázquez et al. (2014). One way in which it is being used is to capture the perception of the inner state of a participant and the value he/she attributes to the interaction, as in the case of Castellano et al. (2009). Poggi (2007), for example, defined engagement as “the value that a participant in an interaction attributes to the goal of being together with the other participant(s) and continuing the interaction.” Other works highlight the emotion component more predominantly such as Subramainan et al. (2016a), Choi et al. (2012), and Sanghvi et al. (2011). For example, in Sanghvi et al. (2011), user's engagement with the iCat is characterized by an affective and attention component. Similarly also Youssef et al. (2019) rely on several levels of engagement annotation including both the affective as well as the (non-)verbal cue level that includes head rotation and eye-gaze. Finally, Biancardi et al. (2019a) combines affective- with attention- and cognitive engagement components in their detection model. Every person might of course differ to a certain degree in the way that he/she expresses the different forms of engagement.

Engagement with a task is typically referred to as *task engagement*. There is great variety in definitions of task engagement. On the one hand, task engagement is defined as a human involved in a task (Corrigan et al., 2015). In such a context, the human does not interact with an interface, a robot, an agent to perform the task. Since our focus is on human-agent interaction, we will not consider this case. Rather we will consider task engagement in the context of human-agent interaction where a human and an embodied agent interact together around a task. On the other hand, task engagement can also refer to any kind of human-agent interaction in which behaviors are centered around a task. Examples of such interactions include the one of moving objects, or an object learning experiment where the agent asks participants to identify the name of objects so that it can learn them (Ivaldi et al., 2014), or mobile robots approaching humans (Ramírez et al., 2016).

In recent years there has also been an increasing amount of work going beyond dyadic to group interactions. This includes work on engagement as well. There are different ways to approach quantifying engagement in a group. Gatica-Perez et al. (2005) defined group interest as “the perceived degree of interest or involvement of the majority of the group.” Salam et al. (2017) defined “group engagement” as “the engagement state of two entities in the interaction together with another entity.” Oertel et al. (2011) defined group involvement as “a group variable which is calculated as the average of the degree to which individual people in a group are engaged in spontaneous, non-task-directed conversations.” Similarly, Salam et al. (2017) defined group engagement as “the engagement state of two entities in the interaction together with another entity.” They stress the importance of distinguishing group engagement from other group related constructs such as “cohesion” (Carless and De Paola, 2000) and “mutual engagement.” Goffman (2017), who built on Clark (1996), refers to people within an interaction as belonging to different participation categories. To make this classification, he first distinguishes between participants and non-participants. The group of participants he considers consist of “the speaker,” “the current addressee,” and “the sideparticipant.” The group of non-participants includes the categories of “bystanders” and “overhearers.” This highlights that a group is not a simply a set of dyads.

Finally, engagement is also being investigated within the context of long-term human-agent interaction. In such a context (Trinh et al., 2018) separate between three user categories: Those who “dropout,” those who are “moderately engaged” and those who are “highly engaged.”

As can be seen in Table 1, there is quite some variation in roles taken on by the virtual agent or robot respectively. No clear distinction becomes apparent between roles taken on by the robot or roles taken on by the agent. However, the more recent papers seem to use more often the role of a conversational partner. At the same time many of the recent papers also have an affective component to their engagement definition. A probable reason for this might be that recent developments have lead to great improvements in multi-modal sensing in general and speech recognition in particular, which makes the implementation of mixed-initiative interactions more feasible.

**Table 1:**
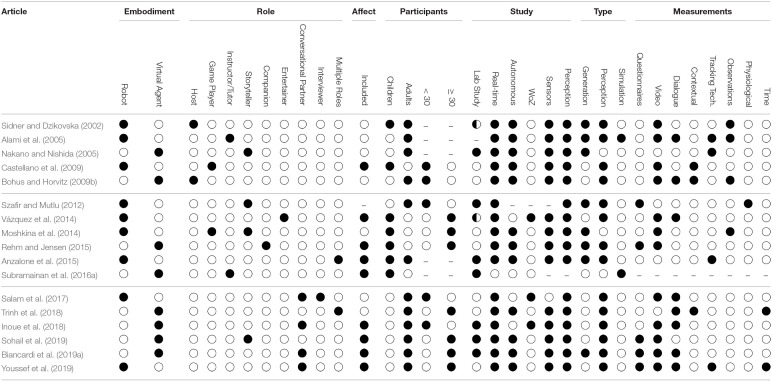
This table illustrates a selected list of articles, based on section 3, to represent our annotation procedure and to provides an overview of the attributes annotated including embodiment, role, affect, participants, study design, type of interaction capabilities, and measurements.

*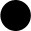, Yes; 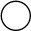, No; “−″. N/A; 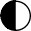, In-between*.

**Table 2:** A summary of the annotation schema categories used.

**Category**	**Description**
Title	The title of the research paper or article
Year	Publication Year
Scenario	A description of the human-robot scenario used in the article
Application	What is the main application domain presented in the article
Robot	Type of robot(s) presented in the article
Role	The main role of the robot/agent with in the scenario presented
Definition	The definition of “engagement” presented in the context of the article
Affect	Is there an affective component to the engagement definition
Participants' type	Are the participants of the study children, adults or both
Number of participants	Indicates the number of participants included in any study (or studies) presented in the article
Lab study	Did the article include a study that is conducted in a laboratory settings
Sensors used perception/how?	What sensors are used to measure engagement (if any)
Perception	Is sensory information used for training an ML model?
Research objectives	What are the aims (purpose), research questions and/or hypotheses presented in the article
Engagement type	Is the article focusing on the perception, generation or simulation of engagement
Study attributes	What are the main evaluated attributes presented in the article
Scenario tag	In what context is the interaction taking place?
Finding/s	The main finding presented in the article
Mode	Was the operating mode of the robotic agent automated or based on a Wizard-Of-Oz approach?
Language	What language was the study conducted in?
Country	Where was the study conducted?
Perception	How is participant perception measured?
measurements	Questionnaires/video analysis/observation
Mode of use	Was the technical setting conducted in real-time, simulated or crowd sourced
Type of paper	What is the main type of the article presented (e.g., Technology, User evaluations etc.)

It can be concluded that there are several definitions of engagement. These definitions are not contradictory but rather complementary. They show that engagement is a multi-faceted phenomenon. However, we also notice that the majority of papers reviewed do not directly define engagement or remain vague in its definition. Similarly, papers do not always make a clear distinction between task and social engagement as the scenarios they use often involve both social and task components. For example, a robot as an assistant is a common scenario. However, the role of an assistant comprises both social and task components.

## 4. Situated Interaction

In the following sections we are referring to engagement as it pertains to interaction in general. In many of the examples given, however, engagement is discussed in the context of conversation. We see and reviewed engagement, however, in general and see conversation as a specific instance of interaction. The fact that conversation as an interaction scenario is represented particularly frequently is an artifact of the papers reviewed.

It appears that situations and scenarios do vary considerably across studies. Examples, of such scenarios include museum guides (Pitsch et al., 2009; Salam and Chetouani, 2015; Biancardi et al., 2019a), games (Díaz et al., 2011; Klotz et al., 2011; Leite et al., 2014), hospitality (Sidner and Lee, 2003; Sidner and Dzikovska, 2005), education (Leyzberg et al., 2014; Papadopoulos et al., 2016), sales (Ishii et al., 2011), and receptionist/direction giving (Michalowski et al., 2006; Bohus and Horvitz, 2014). With regards to museum guides, the overarching goal in the studies has been on making visitors more interested in the artwork and to provide information on the artwork on demand. Such studies have been done both with virtual agents and with robots. These interactions require a knowledge base of the paintings as well as interaction management with the users. Interactions in general are made more difficult by the surroundings in which the interaction is taking place. In a museum generally the noise level is high, participants are passing by in an uncoordinated manner and the number of interaction partners can vary from one to many. This situation puts certain constraints on human-robot interaction, such as the robustness of speech recognition and turn-taking regulation. However, not all interactions rely on dialogue. Some Human-Robot interactions are text-based or picture based. In addition also the degree of interactivity varies. Some systems are based more on providing information on demand whereas others act more interactively (Bohus and Horvitz, 2009b). Other interactions are in contrast being designed for or already situated in a home environment (for example, supporting health-care, or also in the context of a social companion) (Sidner, 2016). Researchers have many purposes. Generally in a context like these there is not so much noise, the environment is quieter and the interaction is more focused on a single user or a smaller group of people. However, the interaction is also required to go more into depth and social aspects are more important. Also, memory and variability of interaction become more essential. One further aspect is that of an experiment being situated within a controlled laboratory environment vs. an open, uncontrolled environment.

### 4.1. Lab or Real World

If we want to go toward more long-term interactions and away from very context dependent interactions, then it is important to gather data and build models of how interactions happen in real-world situations. Questions then arise including how to capture changes of engagement over the course of an interaction, how group size effects engagement and what implications this has on model building. To capture more of these conversational dynamics, Oertel et al. (2013) recorded five participants over the course of two days. All interactions were recorded with audio and video and also motion captured. Instead of providing participants with a task or guiding the interactions, they were simply asked to talk to one another. While this data led to several research papers on investigating engagement, it was also limited in that still several multi-modal cues needed to be annotated manually. Specifically, the engagement label itself but also lower-level cues such as eye-gaze and voice activity still needed to be annotated manually. Oertel et al. (2014) created a corpus which allowed them to study engagement in a setting that fostered free flowing interaction. However, at the same time it was much more controlled in terms of interaction phases. Participants were much more restricted in terms of conversation topic (i.e., their PhD studies) and movement (they were asked to remain seated around a table). Due to this set-up it was however possible to infer gaze and speech activity automatically as well as to optimize for changes in conversational dynamics.

There are more corpora available which try to capture engagement in lab or in the-wild settings to different degrees. Many of them are however not publicly available. Such corpora are then often used in order to predict engagement states of participants, examples include Kim et al. (2016a, 2018), Oertel and Salvi (2013), and Oertel et al. (2011).

In the HRI community, research is going more and more toward bringing robots out of the classical lab environment and evaluating them “in the wild.” The notion of what “in the wild” entails often remains unclear. In the following section we are going to highlight differences between in the wild and lab settings and point toward challenges and advantages associated with each scenario. The vast majority of studies reviewed are lab studies. The “in the wild” studies were mainly concerned with long-term interaction or robot child-interactions or both. Examples of child-robot interaction scenarios often concern museum environments. The manner in which the interaction is then realized can vary widely. For instance, Rehm and Jensen (2015) and Siegel et al. (2009) evaluate engagement in the context of a museum. However, in the former case, the agent is a monster agent which ate artworks and the children had to find information about these cultural artifacts. This interaction is quite different to an experimental set-up which is centered around conversations between an agent and a human. Ahmad et al. (2017), for example, carry out a long-term interaction experiment in which children play a game of snakes and ladders with a Nao robot. The children's engagement is later analyzed offline. Similar to this experiment, also Moshkina et al. (2014) carried out their experiments in a public space but here the difference was that the authors were interested in short-term interaction. That interaction revolved around story telling and the experiment was more concerned with investigating how a change of different multimodal cues of the agent effects the social engagement of the human.

A middle ground for lab or real-world settings is the school setting as for example used in Zaga et al. (2015), Castellano et al. (2017), and Leite et al. (2014). Students are situated in environments they are more familiar with but most school experiments are still pull-out studies. This means that experiments are typically more controlled than if they were carried out in a public space such as a museum. Castellano et al. (2017) look at the effect of task, social context, and their interdependencies in human–robot interaction. Yet, it needs to be noted that engagement is here only evaluated indirectly through an assessment of interaction quality. Castellano et al. (2014), also in a school context, evaluated the effect of robot initiative on the students learning task and the perceived engagement. Similarly, Leite et al. (2014) investigated the effect of an empathetic robot designed for long-term interaction on social presence, engagement and perceived support in children. Similar scenarios are also found in Castellano et al. (2017), Castellano et al. (2014), Leite et al. (2014), and Castellano et al. (2013).

Pitsch et al. (2009) investigated contingent versus non-contingent behavior strategies and the effect on the engagement of the user. This approach is quite different in terms of the scenario than the more game oriented approaches listed earlier; although, also here the task of the robot was to provide information about paintings. Moshkina et al. (2014) investigate the behavior of a robot in a public space. Similar to the previous examples, the scenario is concerned with story telling and game playing and is notable for the large number of participants (around 400) involved in the experiment. Alač et al. (2011) use a game-like interaction to focus on the interaction of a robot and the effect of different activities on the engagement of children. An additional scenario in the wild, conducted by Šabanović et al. (2013), involves studying the effect of robots on participant engagement in nursery homes. Finally, in the wild scenarios also include office environments in which agents take on the role of receptionists (Bohus and Horvitz, 2009b) or direction givers (Bohus and Horvitz, 2014).

### 4.2. Annotations

The way in which engagement is being measured is crucial for its quantification and generalizability across different studies. However, in a similar manner to defining engagement, conducting annotations of engagement is also a challenging endeavor since there are no generally accepted and established annotation schemes in use. With regards to perception studies, there are two general approaches for annotating engagement. The first relies on first-person annotations of the interaction and the second one on third-party observer annotations. First-party observer annotations are generally provided at the session level. While this approach has the advantage that it captures the perception of the participants in the study, it has the disadvantage that it is not well-suited for capturing the conversational dynamics within the session. To capture conversational dynamics within a session, a third-party observer approach using thin-slicing, see for example Ambady and Rosenthal (1993), might be better suited. In a thin-sliced approach, video segments ranging from 5 to 30 s are extracted. These segments of audio and/or video are then being rated by several annotators and inter-rater reliability is calculated. The scale on which engagement is measured also determines how inter-rater reliability measured. There are several ways of conducting the annotation. Some approaches use a binary annotation scheme whereas others use a scalar or ordinal scale annotation schemes. Commonly used inter-rater reliability measures are Cohen's kappa, Fleis' kappa, and Krippendorf's alpha. Regarding the generation of engagement, there does not seem to be an equally established trend. Engagement and disengagement are here often associated with the presence or non-presence of an interaction event.

## 5. Perception

The vast majority of studies reviewed here investigate engagement from a perception system point of view which means that sensors are used as a primary means of input for estimating the degree of engagement or an engagement state. One common task is to estimate a human user's engagement in a conversation or task and to then adapt the robot's behavior accordingly. The purposes and research questions behind it vary widely which is also linked to the issue of differing definitions of engagement. For example, a case in which engagement is defined by the proximity to a robot will have very different implications for sensors and the perception system than in a case where engagement is defined by subjects' participation in a conversation. Another different approach is the perception of engagement through visual attention mechanisms. Yet again, the perception of signals related to engagement is used to evaluate the impact different tasks have on the unfolding of conversations. A further application for the perceptual detection of engagement is long-term interaction. In order to engage a human for a longer period of time, engagement detection and reactions are becoming increasingly important.

### 5.1. Automatic Prediction of Engagement

Several works have addressed the automatic prediction of engagement. In general a distinction can be made between rule-based and machine learning-based approaches for the prediction of engagement. Examples of studies that report rule-based approaches are Brown et al. (2013), Glas et al. (2015), Ishii and Nakano (2008), and Rich et al. (2010). There are differences in how rules are implemented. For example, in Ishii and Nakano (2008), rules are based on gaze-transition patterns, whereas in Brown et al. (2013) they are based on the speed of students' responses to a math test. In many studies however, *social engagement* is measured through eye-gaze (see Nakano and Ishii, 2010; Qureshi et al., 2013), due to the close relationship between *visual attention* and engagement. Ishii et al. (2013a), for example, found that the use of various gaze features provides a good estimate of the user's conversational engagement.

In addition to eye gaze, other measures of user attention have been proposed in the literature. Szafir and Mutlu (2012), for example, designed a system that allows a robotic agent to monitor student attention in real-time using measurements from electroencephalography (EEG).

As far as machine learning-based methods are concerned, while the proposed methods vary to a great extent, many of the studies rely on Support Vector Machines (SVMs) and use eye-gaze or head-pose as input-features for the engagement prediction. Oertel and Salvi (2013) investigated individual involvement and group engagement. They used different gaze variables to first summarize and explain group actions and then to investigate whether changes in these variables are good predictors of engagement. Moving beyond user attention as a measure of engagement, works by Sanghvi et al. (2011) and Castellano et al. (2014) found that patterns of postural behavior can be used to accurately predict the engagement of children with a robot during game play and that the latter can also be predicted using information about the children's valence, interest and anticipatory behavior. In their work, social engagement is modeled as a state consisting of affect and attention components in the context of the interaction. Castellano et al. (2009) also found that an approach that includes both task and social interaction-based features to measure engagement with a robot outperforms those based solely on non-verbal or contextual information. Castellano et al. (2017) showed that game and social context-based features can be used to predict engagement with a robot and that their integration with context-based features encoding their interdependencies leads to higher recognition performances. Kim et al. (2016b) proposed to approach the automatic prediction of engagement using an ordinal learning method and showed that such a method can successfully be used to predict children's engagement using non-verbal features. Foster et al. (2017) used Conditional Random Fields to predict engagement in multi-party HRI using audio-visual data. In their work the task was to estimate engagement of customers for a robot bartender based on the data from audiovisual sensors, which relates to the need for a robot in a dynamic real world environment to infer people's intentions in the scene in order to only attend to those who wish to interact with it. Ishii and Nakano (2010) found that taking into account individual differences of users in gaze transition patterns performs the best in predicting user's conversational engagement. Ishii et al. (2011) then extended their model by adding to gaze transition patterns other gaze parameters such as the occurrence of mutual gaze, gaze duration, distance of eye movement (toward objects of interest in the interaction), and pupil size. They found that considering gaze behaviors in their complexity enhances the performance for predicting user's conversational engagement.

In addition to verbal and non-verbal behavior and context, other modalities, and social variables have been identified as important for the automatic prediction of engagement. Choi et al. (2012), for example, found that people's physiological reactions such as heart rate and electrodermal activity can predict the extent to which people will engage affectively or strategically with an agent. Moreover, Salam et al. (2017) found that taking into account personality for the classification of engagement is important. Similarly, Ivaldi et al. (2017) showed that engagement models classically used in human-robot interaction should take into account attitudes and personality traits.

#### 5.1.1. Deep Learning Approaches

As mentioned in previous paragraphs, machine learning techniques have been widely used for engagement recognition. The success of these techniques heavily depends on both the choice of data representation (input features) and annotation on which they are applied. Most of the input features are domain specific and data representation usually results in a feature engineering phase, as exemplified in Anzalone et al. (2015). The main advantage is the explanatory dimension of the input features. In Leclère et al. (2016), the percentage of time spent face to face or oriented to the task is used to assess face-to-face and task engagement in clinical settings. However, the features are not easily transferable to new tasks, situations and applications. Improving data representation for classifiers is the main objective of representation learning, as described in Bengio (2011). Deep learning is a specific method for achieving representation learning using multiple non-linear transformations. Representation learning based on deep learning is particularly of interest in multimodal processing of human behavior data by reducing the need of priors on the nature of relations between modalities, the dynamics of non-verbal signals, nature of the task and their impact on the prediction of socio-cognitive states such as engagement.

In Rudovic et al. (2019a), a deep learning approach called PPA-net (Personalized Perception of Affect network) is introduced to jointly analyse visual (face and body), audio, and physiology data for the prediction of valence, arousal and engagement in autism therapy. The network is designed with three layers: (i) a feature layer, learning representation of each modality, (ii) a context layer, processing of heterogeneous data and expert knowledge, and (iii) an inference layer, predicting the level of arousal, valence, and engagement. Feature representation learning is performed by Auto-Encoders (AE), which transform signals to a hidden representation. Interestingly the approach allows one to integrate the correlations among modalities into the representation learning. The context layer aims to augment the feature representation with expert's inputs, which are domain specific (mainly the assessment of children). The last layer is a multitask learning phase, which aims to learn child-specific layers for valence, arousal and engagement estimation. Taken all together, this architecture allows learning correlations between modalities, introducing expert knowledge, personalization as well as relations between affective states.

Another strong motivation for deep learning approaches is learning the dynamics between features. Even with the use of explainable features such as head pose, the relationship between the dynamics of such features and engagement is not always straightforward. Explicitly learning the temporal dynamics between the features as the mapping to engagement could be performed by deep learning approaches. In Hadfield et al. (2018), a Long Short-Term Memory (LSTM) neural network is employed to classify engagement of children to the task using pose data. LSTM are recurrent neural networks able to capture the different dynamics of time series and they have been shown to be efficient in sequence prediction problems. These models have been successfully applied to engagement recognition using head movements in Hadfield et al. (2018) and Lala et al. (2017) and facial expression in Dermouche and Pelachaud (2019a). Temporal models such as LSTM and Gated Recurrent Unit (GRU) are compared to static deep leaning approaches as well as logistic regression. The results show that temporal dynamics as well as the observation window and buffer delay are important factors in the performance of classifiers.

All these approaches rely on the availability of engagement annotation. Recently Rudovic et al. (2019b) propose a multimodal active learning approach based on deep reinforcement learning to find the optimal policy for active selection of the user's data. The classification of individual modalities into engagement levels (high/low/medium) is performed by LSTM models followed by fully-connected layers. The output of classifiers are also fed to a Deep Reinforcement Learning agent (Q-function). The agent receives a reward related to its decision: a positive reward is given for correct predictions, and negative rewards is given for incorrect predictions or label requests to human expert. This approach is designed for adapting the engagement prediction model to new tasks, using a minimum number of queries. In addition, as in Rudovic et al. (2019a), the approach also allows multimodal processing and personalization.

### 5.2. Automatic Prediction of Disengagement

While most of the works reported in the literature address the automatic prediction of engagement, examples of systems capable of automatically predicting user disengagement can also be found. Leite et al. (2016), for example, developed an algorithm to predict disengagement in small groups of children interacting with two robot actors playing out interactive narratives around emotional words, using visual and auditory features such as voice activity, smiles, postural and gaze behavior. Bohus and Horvitz (2014) also investigated disengagement. They used proximity, stability and attention persistence in order to estimate the degree of user disengagement. Conversational hesitation markers were used to estimate whether a participant is still interested in continuing to engage in a conversation.

More recently, Youssef et al. (2019) addressed the detection of engagement decrease in users spontaneously interacting with a socially assistive robot in a public space. Recurrent and Deep Neural Networks were used to detect user engagement decrease in real-time based on analysis of user's behaviors such as proxemics, gaze, head motion, facial expressions, and speech.

## 6. Generation

Engagement aware behavior generation can be accomplished through a multitude of strategies, which is also exemplified in the papers reviewed in this article. First of all, it can be noted that behavior generation is dependent on its target audience. Engagement strategies will have to be adapted for children or for people with special needs. Moreover, strategies for behavior generation are also dependent on the conversational contexts. Humans are generally very good at estimating an interlocutor's level of engagement and reacting appropriately. Agents need to learn more explicitly when to engage and when to disengage from a conversation. Strategies to achieve exactly this can be both based on verbal cues as well as audio-visual ones. Concerning visual cues, a very important process is the establishment of joint attention, either guiding the interlocutor's attention toward an object or indicating its shared focus of attention by reciprocating the focus of attention.

Investigating the impact of robot types of autonomy (robot teleoperated by a remote operator vs. autonomous robot) on emotional engagement, Choi et al. (2014) reported that participants felt more social presence to teleoperated robots than autonomous robots. Moreover, participants felt more embarrassment when they were interviewed with teleoperated robots than autonomous, for example Baek et al. (2014) found that participants felt more social presence in the company of a person, than a product or a robot. As in Choi et al. (2014), the authors observed that an autonomous robot is not able to easily invoke social emotions. Short et al. (2010) found that participants displayed a greater level of social engagement and made greater attributions of mental state when playing against the robot in the conditions in which it cheated. Moshkina et al. (2014) reported that the more human-like the robot behaves during story-telling, the more social engagement was observed. However, robot's game-playing did not elicit more engagement than other, less social behaviors.

### 6.1. Adaptation Mechanisms

The following section provides an overview of studies concerned with strategies used for generating behaviors both adapted to conversational context and user group, especially focusing on the adaptation mechanisms used. During an interaction, interlocutors adapt to each other at various levels. Several computational models have been proposed to decide when the robot should display a particular behavior or use a specific conversational strategy to call for user's engagement or increase it.

Several works report how the timing of agent's behavior in relation to user's behavior is important in maintaining the engagement and enhancing user's experience of the interaction. Ishii et al. (2013b) find that the use of probing questions by the engagement-sensitive agent successfully recovers the subject's conversational engagement, changes the gaze behaviors of the participant and elicits more verbal contribution. Sidner et al. (2003) argue that, in robot-user interaction, users are sensitive to the robot's gaze and gestures. They found that a robot's gestures attract the attention of users. The authors also report that users gazing at the object relevant to the conversation at the same time as the robot is a strong sign of user engagement. So detecting user's behavior in relation to the conversation and to the robot's behavior is crucial to gain information about how users participate in the interaction. They also note that the rules for driving a robot's engagement maintenance behavior must be more complex than simply copying users' behavior. Robins et al. (2005) found that the provision of feedback from the robot in a timely manner was important for the interaction as well as the rhythm, timing of movement and turn-taking in general. Xu et al. (2013) report similar results for multi-party interaction. The authors conducted an experiment where a robot interacted with multiple people at once. There were two conditions: the first involved the robot gazing at its main interlocutor and managing the distribution of turn-taking between interactants, while the second involved the robot gazing and managing turn-taking randomly. The results show that when the robot shows engagement-aware behaviors in the first condition, it significantly improved the effectiveness of communication and positively affected users' experience.

While most studies consider gaze behaviors, Cafaro et al. (2016) studied how different interruption types affect the perception of engagement. Interruptions may be cooperative when the interrupter participates to the ongoing conversation by asking for clarification, showing agreement, while they may be disruptive when the interrupter shows disagreement, changes topic of conversation, etc. Cafaro et al. (2016) found that when using a cooperative interruption strategy such as completing the speaker's sentence or asking a clarification question, e.g., to increase affiliation, i.e., liking or friendliness (as opposed to a disruptive one that includes showing disagreement or changing topic of conversation), an interrupter is perceived as more engaged and more involved in the interaction.

Other works propose learning approaches for the agent to increase user's engagement. To this aim, the agent learns how to adapt to user's behavior. Szafir and Mutlu (2012) developed an adaptive robotic agent that employs verbal and non-verbal immediacy cues, such as modulating spoken volume and using gaze, head nodding, and gestures, to regain attention during drops in engagement. The robot acted as an instructor telling stories to students wearing EEG headsets. A trained model to detect reductions in engagement from the EEG data was used to trigger robot's immediacy cues. A robot displaying adaptive behaviors to students' behaviors improved their recall abilities. For female students, their motivation in the education task and rapport with the robot.

Keizer et al. (2014) explored social state recognition in multi-party HRI, with a specific focus on building machine learning methods to determine whether a user within a group is seeking to engage with a robot bartender using a combination of multimodal features. Markov Decision Processes (MDPs) were employed to generate socially appropriate behavior by the bartender robot based on each individual user's engagement.

Pelachaud and colleagues have developed several adaptation mechanisms to control a virtual agent whose aim is to maintain user's engagement during the interaction. The adaptation mechanisms work at three different levels: the nonverbal behavior, conversational strategy, and signal levels. Each of these mechanisms have been implemented in the same agent architecture but affect different modules. Adaptation of the conversational strategies is done within the *dialog module* described in Biancardi et al. (2019a). The adaptation at the non-verbal level is done in the *behavior generation module* of the Greta platform, as described by Biancardi et al. (2019b). Finally, the adaptation at the signal level is done within the *animation module* found in Dermouche and Pelachaud (2019b). During an interaction with a user, the agent will optimize each of the adaptation mechanisms by relying on either reinforcement learning or LSTM methods. Biancardi et al. (2019a) conducted an experiment in a science museum where the agent played the role of a museum guide to validate their adaptation models. The agent that adapted its behavior to maximize user's engagement was perceived as warm by participants, but they did not find any effect of agent's adaptation on users' evaluation of their experience of the interaction. As noted in Dermouche and Pelachaud (2019a), engagement was defined by user's behaviors that included gaze directions, facial expressions, and posture shifts.

Bickmore et al. (2011) found that the use of relational behavior lead to significantly greater engagement by museum visitors. In this study, that had 1,607 visitors participating, engagement was measured by session length, number of sessions, and self-reported attitude, as well as learning gains, measured by a knowledge test.

Other works have explored personalized tutoring from the perspective of affective policy learning: for example, affect-related states such as engagement have been used by Gordon et al. (2016) to build reward signals in reinforcement learning (RL) frameworks to select motivational strategies. Gao et al. (2018) developed an RL framework for robot personalization that allows a robot to select verbal supportive behaviors to maximize the user's task progress and engagement (i.e., positive reactions toward the robot) in a learning scenario where a Pepper robot acts as a tutor and helps people to learn how to solve grid-based logic puzzles.

Some papers explicitly focus on engagement toward a task. For example, Zaga et al. (2015) found that students engaged and focused more on a task (puzzle) when the robot acts as a peer than as a tutor. Brown et al. (2013) reported that engaging with the robot during a computer-based math test showed that, while various forms of behavioral strategies increase test performance, combinations of verbal cues result in a slightly better outcome. Ivaldi et al. (2014) found that whether the robot or human initiates the learning task makes a difference on the pace of the interaction and the reaction to attention cues. When the robot is the initiator of the learning task, the pace of interaction is higher and the reaction to attention cues faster. Whether the robot initiates the interaction does not affect the perceived engagement.

Some studies focused on children interacting with robots. Brown and Howard (2013) monitored students' engagement levels while conducting math exercises in the presence of a robot using interaction features such as speed and validity of submitted answers. When student disengagement was detected, for example when there was inactivity for too long or the student was not challenged enough, the robot would employ verbal and non-verbal behaviors that were found to reduce children's boredom during the education task (Brown et al., 2013). These behaviors could be a combination of socially supportive utterances, backchannels, gaze contact, gestures, and head movements. Leite et al. (2016) developed an algorithm to monitor engagement in small groups of children interacting with two robot actors and trigger disengagement repair interventions when necessary. With the help of elementary school teachers, repair strategies were designed. They include the robots addressing the whole group and making generic comments that imply responsibility of all participants, looking at each of the children in the group, and then generating a verbal comment without targeting any specific child, or directly addressing the child with the highest level of disengagement. They found that participants who received targeted interventions had higher story recall and emotional understanding, and their valence increased over time after the interventions.

## 7. Long Term Interaction

Longitudinal interactions or “long-term interactions” in HRI are defined by several researchers as a set of interactions over several sessions. For example, Leite et al. (2013) address the question on what defines a long-term interaction with a robotic agent. In their work, they state that the main aspects to define a long-term interaction are based on the number of interaction sessions and the duration of each session. Specifically, they suggest that what constitutes long-term interaction is the point in time when the novelty effect wears off.

Adopting the definition by Leite et al. (2013) on long-term interaction, we could find only four papers explicitly investigating long-term interactions and engagement: Ahmad et al. (2017), Leite et al. (2014), Díaz et al. (2011), and Bickmore et al. (2010).

Ahmad et al. (2017) presented a study with children to investigate adaptive capabilities of a robot that can sustain a long-term social engagement when interacting with children. In their work, they designed a study that has three adaptive conditions that include game-based adaptations, emotion-based adaptation, and memory-based adaptation. Their study had 23 school participants (aged 10–12 years) that were randomly assigned to each of the three conditions. Participants were asked to do three recorded sessions (10 min for each session) over a period of 10 days. The authors, thereafter, conducted video based analysis to investigate the participant's facial expression, gaze, verbal interaction, and gestures. Their analysis revealed that the emotional-based adaptive robot maintained a longer interaction compared with the game-based and memory based adaptive robots.

Leite et al. (2014) presented an empathic model that is designed to allow for a long-term interaction. In their work, they present a study with 16 elementary school children (8–9 years) interacting with a robot in a chess game scenario. The aim of their study was to investigate children's perception of social presence, engagement, and support toward the robot over time. Their study extended over five weeks in which each participant had one session per week (average of 20 min per session). The authors collected data from open ended interviews, questionnaires, video recordings, affect sensors, and game play logs. Their analysis revealed that in long term child-robot interactions, a robot endowed with an empathetic model was perceived to support children in a similar manner to the support received from their peers. Furthermore, children found the emotion-based support to be their preferred supportive behavior, echoing the results presented by Ahmad et al. (2017).

Díaz et al. (2011) aimed to build a robot capable of maintaining the engagement of children. The authors presented a study to investigate the social bonds associated with child-robot interaction in order to design for long-term interaction for hospitalized children. In their study, they had two phases, the first was a field study using four robots at a school with 49 children (11–12 years). The second phase had 7 children from the 49 to contribute in a lab study with one of the robots 2 months later. Data collected from both phases include video, questionnaires, observations, interviews, and interaction choices. Their analysis reveals that appearance and performance aspects are important design considerations for long term interaction, in particular, they shape the expectations of children toward interactive behaviors and social processes.

Bickmore et al. (2010), on the other hand, presented two studies on maintaining engagement in a long-term interactions with virtual agents. In the first study, the authors investigated the effect of the agent's dialogue repetitiveness behavior on retention and adherence for elderly persons. Elderly participants were randomly assigned to each of the two conditions (variable dialogue and non-variable repeated dialogue). Participants interacted with the virtual agent 102.32 days on average. The results of the first study revealed a positive effect on the variable dialogue behavior toward long term engagement. The second study presented by Bickmore et al. (2010), looked at the effect of using personal human back stories, i.e., first person compared to third-person narrative dialogue. In the second study participants were assigned randomly to each of the two conditions. Participants conducted the study over different durations (with an average of 28.8 days). The results revealed that participants rated the first-person dialogue of the virtual agent to be more enjoyable and usable, thus leading to higher engagement.

Overall, the presented HRI studies highlight that long term interactions require the support of emotional robotic characteristics, both at the perception level and at the generation level. In addition, adaptive emotional based engagement helps to maintain a long-term interaction.

## 8. Children

Several studies have investigated engagement in child-robot or child-agent interaction. The majority of them address automatic prediction of engagement and adaptation mechanisms in educational and edutainment scenarios.

In Sanghvi et al. (2011), Castellano et al. (2014), and Castellano et al. (2009), Castellano and colleagues proposed computational approaches for the automatic prediction of engagement in children learning to play chess with an iCat robot in a classroom environment with primary school children. They investigated the role that different behavioral and contextual features play in the automatic prediction of engagement. Their work shows that children's affective expressions emerging in a chess play scenario are very subtle and that performance increases for models that include a combination of social interaction and context-based features. Kim et al. (2016a) showed that levels of engagement can be characterized by relative levels between children. Another example is the work by Rudovic et al. (2019a), who addressed the automatic prediction of engagement from videos in preschool children using deep reinforcement learning.

When it comes to adaptation mechanisms, Procter et al. (2016) investigated improving conversational engagement through data-driven agent behavior modification, by adapting to a variety of different student data sources. Szafir and Mutlu (2012) reported that a robot that increases behavioral cues during passages of low student engagement to regain student's attention improved student's recall abilities. Approaches for the automatic prediction of children's disengagement while interacting with a robot in an educational context and disengagement repair strategies have also proposed by Brown and Howard (2013) and Leite et al. (2016).

Other works explored the interrelationships between affect, empathy, synchrony, and engagement. Hall et al. (2006), for example, found that children are able to recognize and interpret affect in synthetic characters and are empathetically engaged with them in specific scenarios. Chaspari et al. (2015) reported that verbal synchrony between children during game play increases as they become more engaged in a speech-controlled robot-companion game. They showed an interaction with the children's level of engagement: more engaged pairs show higher synchrony in word rate, speech loudness and fundamental frequency. Castellano et al. (2013) showed that a robot's ability to perceive and adapt to children's affect has an effect on their perception of the robot in that children perceive the robot as more engaging and helpful.

Finally, a number of studies investigated engagement perception and generation in long-term interaction. Leite et al. (2014) developed an empathic model for child-robot interaction and found that robot empathic behavior had a positive impact on long-term interaction between children and the robot. They found that ratings of social presence, engagement, help, and self-validation remained similar after five weeks of interaction with the robot. Ahmad et al. (2017) proposed adaptation mechanisms to enable a robot to sustain a long-term social engagement when interacting with school children over a period of ten days. In a different context, Díaz et al. (2011) investigated the social bonds emerging in child-robot interaction in order to design for long-term interaction for hospitalized children.

In summary, this literature survey shows that engagement has been extensively studied in the context of child-robot interaction. It shows that models for engagement prediction and generation via adaptation mechanisms need to be tailored to the specific end-users i.e., children. This represents both a challenge and an opportunity, as it highlights the need to focus on the development and the study of the effects of personalized technologies, if long-term interactions with robots are to be achieved in the future.

## 9. Open Questions

Even though many models of engagement have been proposed, there are still important open questions that ought to be addressed. We list a few in this section.

### 9.1. Multi-Party Interaction

Previous computational models of engagement have focused mainly on dyadic situations. Few models have been proposed for group interaction. Interaction in a group can be very complex. Not everyone in the group may be involved in a task, or participate to the discussion with equal interest. One can address the whole group, a specific person in the group, there may be several sub-groups, etc. Often, the participants in a group conversation are clustered into three main classes: speaker, hearer, and over-hearer. In HCI there may be multiple humans and/or multiple robots. It remains a challenge to define engagement in groups given the multiple conformations that a group may have and given the variety of parameters (role, position, relation to name a very few) group members may take. Measuring engagement in multi-party may require considering more features than in dyads to capture the engagement of each individual in the group, or of sub-groups composing the group. Engagement in multi-party may involve more than involvement and emotion component, such as degree of cohesion and collectivity.

### 9.2. Dynamics of Engagement Process

Another challenge in defining engagement is related to the dynamism of the process of engagement. Engagement is not a static variable. It is a process that dynamically evolves between (two of more) members of an interaction. They may go through different phases of engagement, ranging from disengaged to fully engaged. Most existing works have focused on detecting when participants are fully engaged; very few looked at disengagement (see Leite et al., 2016's work for group disengagement). The question of characterizing the different phases of engagement and defining computational model for their detection is still an open question.

### 9.3. Long-Term vs. Short-Term Engagement

A further open question remains on whether long-term engagement and short-term engagement are referring to the same underlying concept or not. There are some obvious differences in the way engagement is conceptualized depending on the duration of interaction. While turn-taking and visual attention appear to be of more importance in shorter-term interaction, variability of generation and social bonds become more prominent in studies related to longer-term interaction. It appears to be logical that short and long-term engagement are related and that there is an intricate interplay between the two. However, we are not aware of any studies that have investigated their relationship so far.

### 9.4. Task vs. Social Engagement

In this article we discussed studies around task and studies around social-engagement. Most of the studies having haven been carried out so far appear to be in the realm of social engagement followed by studies whose scenarios require a combination between the two. This is very likely also an artifact of currently available sensor technology and thereof resulting limitation in scenario design. There is a high probability, however, that the current developments in sensor technologies will enable a much wider range of scenarios in the near future. A likely application domain appear to be in the factories-of-the future where human-agent collaboration around a task will be in the center of attention. Another likely application domain appears to be in education where currently a lot of emphasis is being given to bringing robots to the classroom. This will necessitate a stronger focus on the conceptualization of task engagement and in depth analysis of how social and task engagement are related to one another in a wider range of application scenarios and longer-duration interactions.

### 9.5. Data Annotation

As far as the implementation of systems for the automatic recognition of engagement in human-agent and human-robot interaction is concerned, an open question is how to annotate data corpora to train prediction models. Specifically, identifying an appropriate ground truth remains a challenge. Trends in the automatic affect recognition community have pointed to the need to move toward the automatic prediction of continuous affect (Gunes and Schuller, 2013). Initial work in the direction of annotating continuous signals of engagement or engagement-related variables in videos of human-robot interactions has been conducted (Corrigan et al., 2016). However, open questions remain about the role of engagement-related variables (e.g., user attention toward robot or toward task) and their interrelationships in task-based interactions, where user and robot (or agent) jointly work on a task, and how this affects the annotation process. Moreover, inter-annotator agreement also remains a challenge for research in this area, due to the multifaceted nature of engagement and its definitions as a process or a state.

### 9.6. On-Line Adaptation

Engagement can be considered as a rich cue that could be then employed to adapt or train machine learning and decision-making models. However, developing computational models that are able to adapt on-line to non-verbal cues is a current challenge in machine learning. The few attempts to exploit non-verbal cues for adaptation and learning require the prior definition of a communication protocol (i.e., meaning of non-verbal cues) between the human and the machine (Broekens and Chetouani, 2019). Training adaptive machine learning with non-verbal cues are facilitated by the prior categorization of limited discrete signals such as pointing or stop hand gesture (Najar et al., 2016). The current on-line adaptive models of engagement exploit a similar approach. In Khamassi et al. (2018), the authors develop an on-line adaptation model to changes in human engagement by considering head pose as an estimator of engagement. However, as discussed in this paper, engagement is a complex construct for which adaptation to it will require the analysis of multimodal cues during a longer period of time.

### 9.7. Context Specificity and Scalability

Many existing human(s)-robot(s) interaction models have been designed for a specific scenario where many of the variables defining an interaction are pre-defined such as the role of the robot, the task to be performed, the interaction setting, the culture of the participants, even the type of the robot, or virtual agent. The models are very context-specific. It is not clear if an engagement model that has been framed so specifically can be applied to any other context. The question of scalability is raised. It is not clear whether a model for a given context may be transferred to another one, or should a new model be drawn. In addition, engagement computational models are usually learned for a specific context and task with a given agent either virtual or physical. How to generalize or transfer such engagement models to other situations is an open-question which in turn not only leads to the problems of defining engagement, data collection and annotation, but also how humans engage in complex interactions with those artifacts. The embodiment of the agent plays a critical role in the nature and quality of those interactions.

### 9.8. Complex Real World Scenarios

A further open question is how to continue to extend engagement research to agents capable of handling more complex, real-world scenarios, which require a blend of social, task and environment engagement. These may be multi-party, multi-task interactions and may involve the agent moving or manipulating the environment, thus requiring it to maintain a greater knowledge of aspects that are often considered outside the context of the interaction in current scenarios. Fundamentally, engagement has relationships to attentive processes and states. As the complexity of interactions increases, more robust foundations may be needed to address these issues. For example, computational visual attention frameworks represent a generic way of resolving the allocation of processing resources across multiple social stimuli, tasks and unexpected encounters in the greater environment in a continuous and flexible manner. Currently in many research studies, the potential foci of attention of the agent are restricted to at most two aspects: the task and the human interactor. A more detailed approach is desirable if more realistic scenarios are to be considered, especially those that are to be robust to the complex, uncertain, dynamic environments in the real world in which important interruptions can take place at any time. For example, one can consider the difficulty of modeling a mobile social agent that is to move down a street in a formation of people. Such an agent would need to attend to unexpected obstacles in order to safely avoid them and maintain its formation while engaging in a socially appropriate manner with the others in the group. These activities would likely lead to many differences in behavior (and in behaviors the agent would be expected to produce) when compared to those observed in contemporary scenarios in which the agent is often static in the environment and facing the interlocutor when the interaction commences.

## 10. Conclusion

This article reviewed studies on engagement within the area of human-agent interaction. It can be concluded that there exists a wide range of definitions. In general, distinctions can be made between studies that treat engagement as a process variable and studies that treat engagement as a state variable. Also the emphasis on task or social aspects of engagement varies widely. In the vast majority of cases the distinction is not made transparently.

A distinction can be made between studies focusing on the (automatic) perception of engagement behaviors and those that focus on the generation of engagement relevant behaviors. Studies reviewed show that both adapting to a target group but also to conversational context is essential. In this article we provide examples of adaptation mechanisms used. Examples of such mechanisms include the use of probing questions or adapting in terms of verbal and non-verbal behavior to the user. While most approaches are rule-driven, there are also some approaches that use machine learning for adaptation, including reinforcement learning and social-state recognition. While several adaptation approaches have been explored, they vary widely in approach and scenario chosen. Due to a lack of benchmarks, a more detailed comparison at this stage does not appear to be feasible.

In terms of the automatic perception of engagement, studies can be divided into two main groups. Those studies which use rule based approaches and those studies which use machine learning based approaches. While the vast majority of studies that are concerned with the automatic prediction of engagement still use traditional machine learning techniques and are mainly SVM-based, there are also a number of recent studies that use deep learning based approaches. Finally, instead of focusing on detecting binary engagement, or different degrees of engagement, there are also a number of studies that focus on detecting disengagement instead.

Regarding robot-child interaction the following conclusions can be drawn. First of all, all studies focus on different aspects of the interaction. These aspects include for example the robot's reaction toward the children's engagement state. This can, but does not necessarily have to be linked to their performance, for example in the context of education. It also seems important that the models for engagement prediction and generation via adaptation mechanisms need to be specifically tailored to children. However, there is still a need to explore the aforementioned point more extensively for definite conclusions. This represents both a challenge and an opportunity, as it highlights the need to focus on the development and the study of the effects of personalized technologies, if long-term interactions with robots are to be achieved in the future. In the studies reviewed no direct comparisons are made to adult-robot interaction, rather the reaction of the children toward the robot were in the center of attention.

Regarding engagement designed for long-term interaction, not many studies have been carried out yet. There is also no clear-cut definition of what constitutes long-term interaction although Leite et al. (2013) suggest that it is the point in time when the novelty effect wears off. Emotional adaptation, appearance and performance, variable dialogue and first-person narrative all appear to be contributing positively to long-term interaction.

More and more work is also concerned with “in-the-wild” studies in contrast to lab studies. Challenges associated with “in-the-wild” studies are that it is much harder to control the interaction. For example noise and suboptimal light sources can interfere with the sensors; the context of the interaction may vary a lot as the number of participants interacting with the robot. The papers reviewed in this article address these challenges in different ways. Some papers focus on collecting corpora which portray non-task-directed interaction to best model engagement dynamics, whereas other papers directly focus on creating an interaction scenario and test it “in-the-wild.” Common scenarios appear to be information-giving, story-telling and game-like interactions. A middle ground between the lab and completely “in-the-wild” location appears to be a pull-out-study in a school setting. Students remain situated in a familiar environment, yet noise level and number of participants etc. can be controlled by the experimenter more easily.

In summary, this review covers a broad range of studies on engagement in human-agent and human-robot interaction. To the best of our knowledge, it is the first review on engagement research that reports on how the human-agent and human-robot interaction communities have addressed issues and challenges relating to engagement definitions and implementations in different interaction settings, engagement annotation, and automatic engagement prediction and generation in adaptive human-agent interactions. The picture that emerges is one of engagement as a highly complex phenomenon that permeates human-agent interaction and determines its success over sustained periods of time. We review open questions and challenges for the community, offering the reader a starting point for making new, interesting research contributions in a research area that is still growing.

## Author Contributions

CO wrote the initial draft of this article which included data analysis, the creation of an initial paper structure, and lead the research discussions on iterative paper improvements. Together with MO, she created the annotation scheme and went on to carry out most of the annotations. GC, CPel, and CPet have initiated discussions on an overview paper on engagement in human-agent interaction, provided feedback on the paper's structure, and written sections of the paper. CPel and CPet also assisted in the paper annotation process. JN helped with the annotation of the data and was involved in discussions on structure and content. MO contributed to the discussions and the development of the annotation scheme, conducted parts of the data annotations and data analysis, written sections of the article, gave feedback on the article's overall structure, and revised the article. MC contributed to the structure, data analysis, writing, and discussions. All authors contributed to the article and approved the submitted version.

## Conflict of Interest

The authors declare that the research was conducted in the absence of any commercial or financial relationships that could be construed as a potential conflict of interest.

## Footnotes

^1^MIT Media Lab Relational Agents, https://affect.media.mit.edu/projectpages/relational/.
